# Neuronal Activity during Exposure to Specific Phobia through fMRI: Comparing Therapeutic Components of Cognitive Behavioral Therapy

**DOI:** 10.3390/life12081132

**Published:** 2022-07-27

**Authors:** Ascensión Fumero, Rosario J. Marrero, Teresa Olivares, Francisco Rivero, Yolanda Alvarez-Pérez, Carmen Pitti, Wenceslao Peñate

**Affiliations:** 1 Departamento de Psicología Clínica, Psicobiología y Metodología, Facultad de Psicología, Universidad de La Laguna, 38200 La Laguna, Tenerife, Spain; rmarrero@ull.edu.es (R.J.M.); tolivarp@ull.edu.es (T.O.); or friverop@ull.edu.es (F.R.); wpenate@ull.edu.es (W.P.); 2 Instituto Universitario de Neurociencia (IUNE), Universidad de La Laguna, 38200 La Laguna, Tenerife, Spain; 3 Facultad de Ciencias de la Salud, Universidad Europea de Canarias, 38300 La Orotava, Tenerife, Spain; 4 Servicio Canario de la Salud, 38004 Santa Cruz, Tenerife, Spain; yolanda.alvarezperez@sescs.es; 5 Dirección General de Salud Pública, Servicio Canario de la Salud, 38006 Santa Cruz, Tenerife, Spain; cpitgon@gobiernodecanarias.org

**Keywords:** cognitive behavioral therapy, exposure, breathing, self-verbalization, fMRI, specific phobia

## Abstract

Cognitive behavioral therapy (CBT) packages for anxiety disorders, such as phobias, usually include gradual exposure to anxious contexts, positive self-verbalizations, and relaxation breathing. The objective of this research was to analyze the specific neural activation produced by the self-verbalizations (S) and breathing (B) included in CBT. Thirty participants with clinical levels of a specific phobia to small animals were randomly assigned to three fMRI conditions in which individuals were exposed to phobic stimuli in real images: a group underwent S as a technique to reduce anxiety; a second group underwent B; and a control group underwent exposure only (E). Simple effects showed higher brain activation comparing E > S, E > B, and S > B. In particular, in the E group, compared to the experimental conditions, an activation was observed in sensory-perceptive and prefrontal and in other regions involved in the triggering of emotion (i.e., amygdala, supplementary motor area, and cingulate gyrus) as well as an activation associated with interoceptive sensitivity (i.e., insula and cingulate cortex). According to the specific tool used, discrepancies in the neural changes of CBT efficacy were observed. We discuss the theoretical implications according to the dual model of CBT as a set of therapeutic tools that activate different processes.

## 1. Introduction

Cognitive behavioral therapy (CBT) is a well-established and useful clinical psychological treatment that has shown efficacy and efficiency for most mental disorders [1,2]. CBT has been recommended as a first-choice treatment for mental health problems of low and moderate severity such as anxiety and related disorders [3,4,5].

In fact, CBT packages for anxiety disorders usually include psychoeducation, exposure to anxiety situations, cognitive restructuring, self-monitoring, self-verbalizations, and relaxation training [6]. The choice of these components mainly depends on the nature of the anxiety disorders [7]. Remarkable results have been obtained with CBT in adults as well as children and adolescents [8,9,10]. Yet, there is still a debate about the role and effectiveness of the various CBT components in clinical practice for treating anxiety problems [11,12].

The reasons why CBT works and the processes it involves are controversial. CBT interventions include cognitive techniques—mainly reappraisal of anxiety stimuli—and behavioral techniques, such as gradual exposure to better manage anxiety. Cognitive restructuring has been assumed to be the key underlying mechanism of CBT efficacy by generating a change from a negatively affected interpretation to a more adaptive one. By contrast, gradual exposure is associated with extinction and inhibitory learning processes [13,14] and is affected by the context of exposure [15]. Moreover, relaxation (i.e., diaphragmatic breathing and progressive muscle relaxation) or self-monitoring may help individuals to learn a way of regulating their physiological stress reactions, increasing their sense of self-control and self-regulation [16].

Studies of neural correlates focused on anxiety disorders have tried to identify the brain areas involved in CBT efficacy. The underlying mechanisms are supported in the so-called dual-path processing model [17], which suggests functional changes in the frontal and limbic brain areas as a result of the efficacy of CBT in phobic disorders. More specifically, CBT produces an increase in emotional regulatory areas (i.e., lateral and ventromedial prefrontal areas) and consequently a decrease in limbic areas, particularly the amygdala. However, data provided by neuroimaging studies about neural changes produced by CBT are more complex than the dual model [18]. A systematic review about the changes associated with CBT in mental health disorders has found a decrease in the activity of the default mode network [19]. This network includes the precuneus, posterior cingulate cortex, inferior lateral parietal gyrus, anterior cingulate cortex, and medial frontal cortex and is responsible for introspection processes, vigilance, and preparation for changes in the external environment. These findings do not support the dual model, since some studies have shown the activation of prefrontal areas, but this activation does not always entail a deactivation of limbic areas [20]. Some researchers have also found an altered function of the anterior cingulate gyrus and precuneus, which are areas usually associated with pre-limbic emotional regulatory processing and intentional behaviors [19]. Similarly, Picó-Pérez et al. [18] found that CBT in anxiety-related disorders activated associative areas, such as the dorsomedial prefrontal cortex, anterior cingulate gyrus, right inferior frontal gyrus, and anterior insular cortex. However, areas associated with fear and anxiety, such as the amygdala, were not significant predictors of CBT efficacy.

Functional magnetic resonance imaging studies about CBT efficacy in anxiety and related disorders do not seem conclusive about the dual-path model and the underlying mechanism of CBT. Neural correlates may differ between studies due to the various techniques included in the CBT package. Different therapeutic components of CBT may activate different neural substrates, which would indicate that there is more than one mechanism explaining CBT efficacy. Some studies suggest that exposure to phobic stimuli can be altered by contextual conditions, such as proximity to stimuli or real or virtual images [15,21]. These different conditions imply changes in brain activation or deactivation areas and in turn affect CBT efficacy [22].

Unfortunately, there is not enough evidence supporting the efficacy of each therapeutic component of CBT, and it is not clear which is the underlying mechanism involved in the efficacy of these different components. The main objective of this study was to analyze the role of two CBT components in phobia to small animals: relaxation through diaphragmatic breathing to reduce the physiological response and positive self-verbalization to promote cognitive processes aimed at providing emotional protection compared to mere exposure. In particular, the goal was to identify the brain activation patterns and various underlying mechanisms through fMRI in each component used. Based on Straube [23], the first hypothesis was that the exposure-only group, when confronted with the feared stimulus, would have higher activation in neuronal networks involved in fear, such as the insula and ACC, compared to the other two experimental conditions. The second hypothesis was that both CBT components (i.e., self-verbalization and breathing) would similarly decrease the activation of brain areas involved in the fear circuit, based on the fact that self-induced intentional distraction and the decision to activate a regulatory strategy has been shown to attenuate activation in areas associated with fear [24].

## 2. Materials and Methods

### 2.1. Participants

Participants were recruited through a public call from the University of La Laguna (Tenerife, Spain). The sample consisted of 30 adults (mean age 21 years, SD = 1.89, 20% male). All participants were exposed to real images of small animals according to their phobia (i.e., cockroaches, spiders, lizards, or rats) and were randomized to one of two intervention conditions (i.e., self-verbalization training –S– or breathing training –B–) or the exposure-only condition –E–. More specifically, 9 participants received verbalization training, 10 received breathing training, and 11 were assigned to the exposure-only condition.

The Inclusion criteria were the following: (a) adults 18 years of age or older; (b) a score of at least 30 in the S–R inventory for cockroaches, spiders, lizards, or rats; (c) not receiving psychological or pharmacological treatment for the phobia; (d) being right-handed; (e) no comorbidities with other mental problems; (f) no vision difficulties that prevented participants from clearly observing the phobic stimuli; and (g) no medical conditions or metallic implants incompatible with MRI.

### 2.2. Instruments

The Composite International Diagnostic Interview (CIDI), Version 2.1 [25], was used to verify the diagnosis of phobia. For the purposes of this study, questions related to a specific phobia, social phobia, agoraphobia, and panic attacks were selected.

The S–R (Situation–Response) Inventory of Anxiousness [26] was administered to obtain a participant phobia rating at baseline. It is a 14-item inventory with a 5-point Likert-type scale that evaluates the most frequent symptoms (i.e., physiological, cognitive, and behavioral) associated with the response to an anxiogenic stimulus (i.e., cockroaches, spiders, lizards, or rats). The inventory has shown high internal consistency (0.95) and adequate convergent validity [27].

The Disgust Propensity and Sensitivity Scale-Revised (DpSS-R-12) [28] is a 27-item scale with a 5-point Likert response format that assesses the frequency of disgust experiences (Disgust Propensity) and the emotional impact of such experiences (Disgust Sensitivity). For the interest of this study, the Disgust Sensitivity scale was administered.

The Hamilton Anxiety Rating Scale (HARS) [29] is a 14-item clinician administered scale with a 5-point Likert-type scale ranging from 0 (not present) to 4 (very severe) that assesses the severity of each anxiety symptom. The scale showed good interjudge reliability, as the intraclass correlation coefficients range from 0.74 to 0.96 [30].

Hand preference was assessed with the Edinburgh Handedness Inventory (EHI) [31] to determine that all participants were right-handed.

### 2.3. Design

We performed an fMRI study with a GE 3.0T Signa Excite HD device to compare three primary measurements with three levels each: self-verbalization, breathing training, or exposure-only. Participants were exposed to a block presentation of videos with real images of phobic stimuli (i.e., spiders, cockroaches, lizards, and rats) in motion filmed in 3D video and neutral stimuli (i.e., wooden balls). The duration of each block was 20 s.

### 2.4. Procedure

The study was conducted from January 2020 to May 2021. After recruitment, an email was sent to potential participants to administer the questionnaires. Based on their scores, a semi-structured interview was conducted online to corroborate the initial diagnosis of specific phobia. Participants who did not meet the inclusion criteria (or met the exclusion criteria) were excluded. Those who met the criteria were assigned a correlative number in order of arrival, and then participants were randomly distributed to one of the three groups, following the previously determined assignment tables. Sex was taken as the stratum, with an assignment table for males and another one for females. The participants of the intervention conditions were trained in self-verbalizations or breathing according to their randomization and instructed to practice these techniques during the following seven days. In the self-verbalization condition, participants were given a list of general adaptive thoughts on spiders, cockroaches, lizards, or rats related to the discomfort that they felt; they were asked to choose 2–3 self-verbalizations and repeat them until they internalized them. The breathing training consisted of taking deep breaths for three minutes.

All participants (i.e., intervention and exposure-only conditions) were scheduled for an fMRI one week after the interview. Participants in the intervention conditions were instructed to apply the self-verbalization or breathing techniques during the fMRI test. This study was approved by the Ethics Committee for Research and Animal Welfare of the University of La Laguna (CEIBA2013-0086).

### 2.5. fMRI and Data Analysis

The brain images were taken from the WFU Pickatlas 3.0.5b [32] (probability threshold 0.5) for SPM12 with the Automated Anatomical Labeling (AAL2) brain atlas [33]. Stimuli were recorded in 3D and projected in the MRI scanner in stereoscopic 3D video using Visual Stim digital MRI-compatible 3D glasses (graphics card: GeForce 8600GT). The Blood-Oxygenation-Level-Dependent (BOLD) signal [34] was used to analyze the neuroimaging measures obtained through the fMRI sessions according to the type of instruction (i.e., self-verbalization, breathing training, or exposure-only). The image dimensions were 4 × 4 × 4 mm voxels, and activation was considered when the activated area was equal to or greater than a 3-voxel cluster (k). The Family-Wise Error (*p* < 0.05 FWE corrected) correction was used. However, when the uncorrected criterion of *p* < 0.001 and k > 3 was 192 mm^3^, activation was considered.

The brain activations were compared using a two-factor ANOVA with the between-group factor experimental condition (self-verbalization, breathing, and exposure-only) and the within-group factor stimulus (phobic vs. non-phobic). A post hoc t-test was calculated to compare the levels in each factor between conditions (i.e., comparing experimental conditions two by two) and within conditions (i.e., phobic vs. non-phobic stimulus). Contrasts of interest in brain regions involved in phobias were performed within a predefined mask. Specifically, the amygdala, insula, and frontal gyrus were masked to test the activation pattern in each experimental condition.

## 3. Results

A significant stimulus × experimental condition interaction (F(2, 54) = 15.28, *p* < 0.05 FWE) was found, with enhanced activation associated with fearful stimuli in the experimental conditions.

### 3.1. Condition Effects

**Figure 1:**
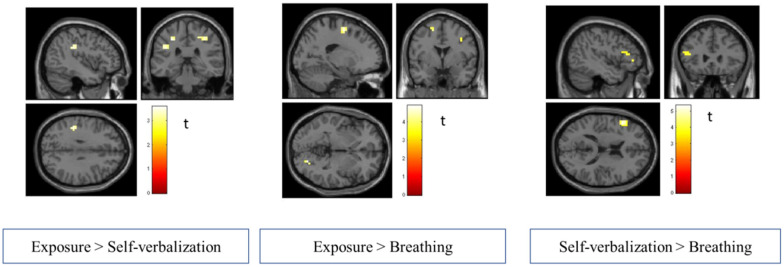
Results of the whole-brain contrast analysis of the exposure > self-verbalization, exposure > breathing and self-verbalization > breathing in cutting planes of the sagittal, coronal, and transversal views.

Simple effects according to the experimental condition showed higher brain activation comparing exposure-only versus self-verbalization (E > S), exposure-only versus breathing (E > B), and self-verbalization versus breathing (S > B). Table 1 presents these effects. We found an activation in the bilateral postcentral gyrus and left supramarginal gyrus involving somatosensory areas in the exposure-only condition compared to the self-verbalization condition. Brain activations of the bilateral superior and middle frontal gyrus, right precentral and fusiform gyrus, and left superior occipital cortex, which are associated with motor and prefrontal areas and emotion regulation, were stronger in the exposure-only condition compared to the breathing condition. In addition, an activation of the ventromedial prefrontal cortex was observed in the exposure-only condition compared to the breathing condition (see Figure 1).

**Figure 2:**
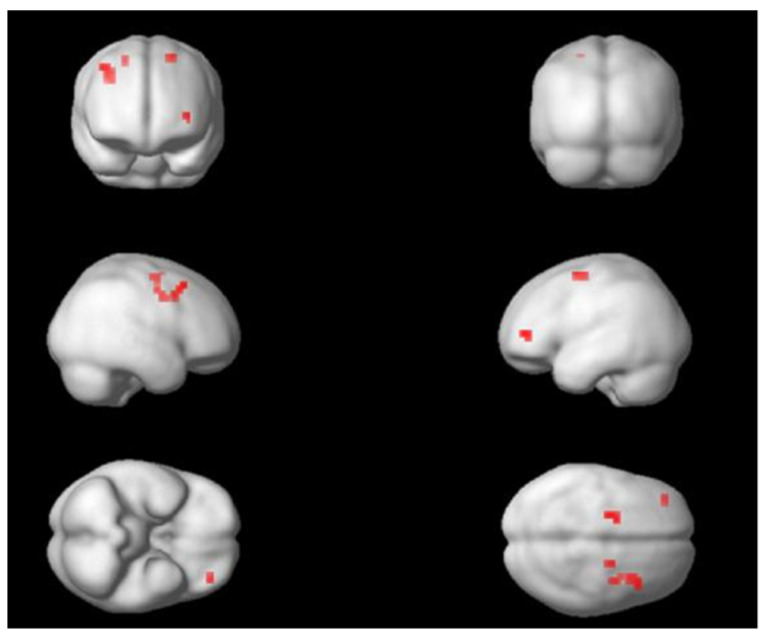
Frontal activation during the exposure > breathing. Red marks show activated areas.

An additional masking analysis comparing exposure-only and breathing conditions was performed (see Figure 2). In the E > B condition, an activation emerged in the left supplementary motor area (peak coordinates MNI x, y, z: −14, −4, 62; k = 3; t = 3.53; z = 3.06; *p* < 0.001 uncorr). The anterior left cingulate also showed a slight activation (peak coordinates MNI x, y, z: −14, 44, 14; k = 2; t = 2.96; z = 2.65; *p* < 0.004 uncorr). The self-verbalization versus breathing contrast showed that the left inferior frontal gyrus (i.e., pars triangularis and pars opercularis) and left middle frontal gyrus were activated. No significant differences were found in brain activation for the remaining contrasts (S > E; B > E and B > S).

### 3.2. Masking Effects

**Figure 3:**
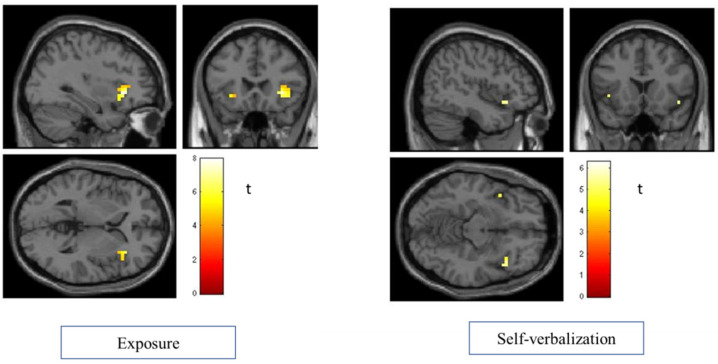
Results of the an inclusive mask analysis of the insula in the exposure and self-verbalization conditions.

In the self-verbalization condition (see Figure 3), a cluster emerged in the right insula (peak coordinates MNI x, y, z: 38, 20, −6; k = 8; t = 5.56; z = 3.46; *p* < 0.0003 uncorr; x, y, z: 46, 20, −10; t = 5.30; z = 3.38; *p* < 0.0004 uncorr), and an activation in the left insula was observed (peak coordinates MNI x, y, z: −46, 12, −6; k = 6; t = 6.28; z = 3.67; *p* < 0.0001 uncorr). No activation in the insula was found in breathing.

**Figure 4:**
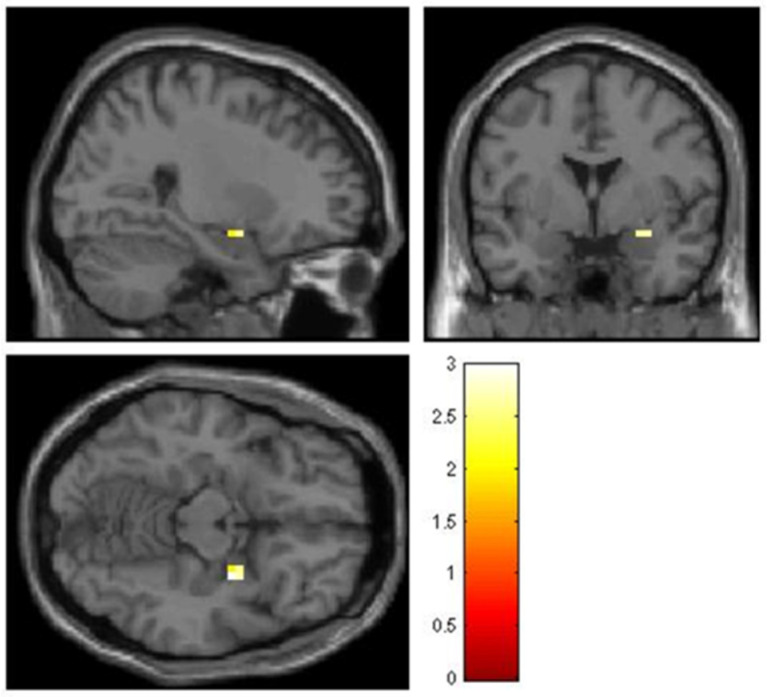
Results of the an inclusive mask analysis of the amygdala in the exposure condition.

Activation in the amygdala was found in the exposure-only condition (peak coordinates MNI x, y, z: 30, −4, −14; k = 4; t = 2.99; z = 2.47; *p* < 0.0068 uncorr) but not in the self-verbalization or breathing conditions (see Figure 4). In the exposure-only condition, a cluster emerged in the right insula (peak coordinates MNI x, y, z: 34, 24, −2; k = 38; t = 7.96; z = 4.37; *p* < 0.00 uncorr; x, y, z: 38, 20, −10; t = 6.95; z = 4.11; *p* < 0.00 uncorr), and an activation in the left insula was observed (peak coordinates MNI x, y, z: −30, 24, −6; k = 3; t = 5.18; z = 3.53; *p* < 0.00 uncorr).

### 3.3. Stimulus Effects

Simple effects of the stimulus were explored in each condition. In the exposure-only condition, an activation in the right thalamus was observed (peak coordinates MNI x, y, z: 22, −28, −2; k = 5; t = 10.58; z = 4.90; *p* < 0.0000 FWE) during exposure to phobic stimuli. In the self-verbalization condition, we observed an activation in a cluster composed of the right cerebellum, fusiform gyrus, and inferior occipital lobe (peak coordinates MNI x, y, z: 34, −60, −22; k = 35; t = 23.91; z = 5.73; *p* < 0.0000 FWE; x, y, z: 34, −68, −14; t = 23.26; z = 5.69; *p* < 0.0001 FWE; x, y, z: 38, −80, −10; t = 21.19; z = 5.57; *p* < 0.0002 FWE, respectively) and a cluster composed of the left cerebellum and fusiform gyrus (x, y, z: −26, −56, −22; k = 8; t = 15.00; z = 5.08; *p* < 0.0037 FWE; x, y, z: −34, −64, −18; t = 15.83; z = 5.16; *p* < 0.0024 FWE) and left inferior occipital lobe (x, y, z: −38, −72, −6; k = 5; t = 18.06; z = 5.34; *p* < 0.0009 FWE). In the breathing condition, we observed an activation in the left middle and inferior occipital lobe cluster (x, y, z: −34, −80, −2; k = 18; t = 15.57; z = 5.36; *p* < 0.0008 FWE; x, y, z: −30, −76, −6; t = 12.46; z = 5.00; *p* < 0.0055 FWE, respectively) and the right lingual gyrus (x, y, z: 26, −84, −10; k = 5; t = 13.84; z = 5.18; *p* < 0.0018 FWE).

## 4. Discussion

This study compared the effects of exposure to phobic stimuli of small animals in three experimental conditions (i.e., self-verbalization, breathing, and exposure-only). The first hypothesis was supported, since the exposure-only condition showed a stronger activation of neuronal networks involved in fear of the phobic stimulus compared to the other two experimental conditions, in which adjuvant intervention techniques were applied. On the other hand, both intervention techniques showed their efficacy by producing a decrease in the activation of the brain areas associated with fear, although the brain regions activated were not the same, partially supporting the second hypothesis. Neuroimaging techniques provide neurobiological support for the efficacy of CBT in the treatment of phobic disorders [35,36].

Overall, the exposure-only condition showed a stronger activation in frontal areas compared to the other experimental conditions. Specifically, the exposure-only condition exhibited greater activation in the superior and middle frontal gyrus, precentral gyrus, and superior occipital cortex compared to the breathing condition. Additionally, an activation in the ventromedial prefrontal cortex, supplementary motor area, and anterior and middle cingulate gyrus was found but with a smaller size. The exposure-only condition also showed greater activation in the postcentral gyrus and supramarginal gyrus compared to the self-verbalization condition.

Therefore, the condition of participants in the exposure-only condition showed an activation in the brain areas involved in fear, both at the sensorimotor, visual, and attentional levels, and in superior cognitive functions. This suggests that exposure participants require a greater effort to regulate their emotions upon presentation of the phobic stimulus; this is probably linked to the compensatory mechanism associated with the severity of the phobic stimulus [37]. Previous research has shown that a brief exposure to spiders in persons with phobia activated the areas involved in fear, such as attention, visual areas, subcortical fear, and higher-order language, even if the participants were unaware of the fear [38]. Along the same lines, a systematic review found that subliminal exposure to specific phobias produced an extinction of the physiological and behavioral responses of fear, although the subjective experience of fear was barely modified [39].

When analyzing the activation of areas associated with fear, such as the insula and amygdala, it was observed that the insula was activated in both the exposure-only condition and the self-verbalization condition, but with a greater intensity and size in the exposure-only condition. These findings are confirmed by previous research that showed the role of the insula in emotion processing [40,41]. Results suggest that in the self-verbalization condition, participants remain aware of the feared stimulus, since they are paying attention to it and trying to tell themselves that “nothing is happening”. Positive self-verbalizations in children with social phobia and generalized anxiety has been contraindicated because dysfunctional thoughts are interrupted, and the children cannot realize their fears and perform a reality check [42]. However, other studies indicate that to treat spider phobias, ignoring the stimulus and trying to remain calm reduces activity in the visual cortex in response to spider pictures, so that the planned response overcomes the usual fear response [43].

As far as the amygdala is concerned, it only showed significant activation in the exposure-only condition in the presence of the phobic stimulus. These results confirm a higher emotional value linked to the sensory experience. This seems to suggest a greater effort (i.e., cognitive resources) to regulate the emotions associated with the presentation of phobic stimuli when there is no strategy to deal with them. Other studies have found that the frontal regions engaged by cognitive emotion regulation strategies may inhibit the amygdala through ventromedial prefrontal cortex connections [44]. Similarly, Åhs et al. [45] observed that visually elicited phobic reactions deactivate prefrontal areas involved in cognitive control over emotion-triggering areas such as the amygdala, resulting in motor readiness that implies support for fight or flight behaviors.

Direct contrasts of self-verbalization and breathing indicated a reduced activation of the areas involved in fear, suggesting that both techniques may have had beneficial effects on emotional regulation of fear, acting as an “anxiolytic” resource. Yet, a greater activation appeared in the pars opercularis and pars triangularis in the self-verbalization condition. These types of techniques may operate because the attention focus is manipulated by requesting participants to apply the previously trained adjuvant technique. The pars opercularis and pars triangularis could be involved in emotion regulation and contribute to the top-down control of negative emotions [46]. Psychotherapy has been shown to work at a top-down level by developing meanings to regulate cognitive processes that reduce the emotional dysregulation associated with the limbic functions [47]. Other studies have demonstrated the efficacy of interventions based on exposure to phobic stimuli that combine subliminal exposure (i.e., reducing behavioral and physiological responses) and supraliminal exposure (i.e., reducing the conscious feeling of fear) [39].

When both techniques were compared in the self-verbalization condition, an activation of the areas involved in the top-down emotional regulation of negative emotions with a high level of arousal (operculum-insula) was maintained. By contrast, in the breathing condition, a reduced activation of the fear network regions was observed. This finding could be explained on the basis of the reciprocal inhibition process [48]. Breathing involves an automatic physiological response that is incompatible with the anxiety experienced during feared stimulus presentation. This adjuvant technique may generate a state of relaxation that inhibits the brain activation of the anxiety antagonistic response. Diaphragmatic breathing and relaxing have proven to be an effective strategy against stress and different types of phobias. Nevertheless, it has also been found that individuals with a higher intensity of fear are unable to maintain exposure during relaxation treatment [49,50]. Both techniques share an activation of the direct antagonist of anxiety states, directly related to limbic and paralimbic areas, but they fail to activate emotional regulation in prefrontal and ventromedial areas. In this regard, self-verbalization and breathing do not participate in the activation of the dual-path model for CBT efficacy, as has been formulated [17].

Some limitations of this study refer to the fact that due to the small size of the experimental conditions, it was not possible to analyze each specific phobia separately. Another limitation is that although the participants showed high levels of anxiety when facing phobic stimuli, no assessments indicated the severity of the phobia. In addition, records of brain activation were not taken before applying the experimental manipulation but later during the application of the intervention technique, so the effects of each technique in reducing fear are unknown. In the future, it would be interesting to evaluate the changes that occur before and after an experimental manipulation in which this or another type of therapeutic strategy is applied. Likewise, it would be a good idea to consider applying an intervention program in which both techniques are combined to compare the joint and/or separate efficacy of each in reducing fear. Moreover, the effects of these intervention techniques in other behavioral (i.e., stimulus avoidance) and subjective (i.e., self-informed fear) correlates could be explored.

In summary, the findings confirm the usefulness of positive self-verbalization and breathing in controlling anxiety activation. These two adjuvant techniques are widely used in the CBT package for anxiety disorders. Specifically, participants who did not undergo an intervention showed a higher activation in areas that imply giving a higher emotional value to the stimulus (i.e., the amygdala, ventromedial prefrontal cortex, supplementary motor area and cingulate gyrus) and a higher subjective experience of the emotion (i.e., the insula and cingulate cortex). The effects of the therapeutic strategies on the neural correlates of fear indicated that there was no significant activation of the areas linked to the processing of phobic stimuli, suggesting that both strategies were effective in regulating the emotion of fear, mainly breathing, acting as antagonists of anxiety activation processes. Yet, the fact that these adjuvant techniques do not reproduce the dual model explaining CBT efficacy may contribute to the idea that CBT, as a set of therapeutic tools, activates different processes according to the specific tool used. This may also contribute to explaining the discrepancies observed in the neural bases of CBT efficacy [18,19]. These findings have at least two implications: (i) the design of treatment programs will be improved by translating these findings into clinical practice, and (ii) it is necessary to study each part of CBT packages, because they may activate different underlying mechanisms.

## Figures and Tables

**Table 1:** Comparison of effects of self-verbalization, breathing, and exposure on neural processing of phobic stimuli.

AREA	Coordinates	*k*	*Z*	*F*	*p*
Condition X Stimulus					
L Superior frontal gyrus	−18, −4, 58	14	5.92	30.10	0.0000
L Inferior frontal gyrus (pars triangularis)	−50, 20, 18	17	5.28	22.93	0.0000
L Middle frontal gyrus	−38, 48, 6	7	5.23	22.35	0.0000
L Inferior frontal gyrus (pars triangularis)	−42, 40, 2	*	4.71	17.71	0.0000
R Middle frontal gyrus	34, 12, 42	9	5.00	20.15	0.0000
L Superior occipital cortex	−10, −84, 22	5	4.93	19.59	0.0000
R Superior frontal gyrus	22, −4, 58	6	4.78	18.24	0.0000
R Striatum	18, 12, 10	10	4.77	18.15	0.0000
R Precentral gyrus	38, −4, 46	5	4.71	17.71	0.0000
L Postcentral gyrus	−38, −32, 62	3	4.56	16.52	0.0000
Comparison conditions E > S					
L Postcentral gyrus	−30, −32, 46	4	3.07	3.58	0.0011
L Supramarginal gyrus	−42, −32, 30	11	3.00	3.47	0.0014
R Postcentral gyrus	34, −32, 46	10	2.92	3.36	0.0017
R Postcentral gyrus	26, −32, 50	*	2.68	3.02	0.0037
Comparison conditions S > B					
L Inferior frontal gyrus (pars opercularis)	−50, 20, 18	27	4.04	5.35	0.0000
L Middle frontal gyrus	−38, 48, 6	7	3.48	4.28	0.0002
L Inferior frontal gyrus (pars triangularis)	−42, 40, 2	*	3.33	4.03	0.0004
Comparison conditions E > B					
L Superior frontal gyrus	−18, −4, 58	13	3.89	4.89	0.0000
R Fusiform gyrus	22, −76, −2	4	3.87	4.87	0.0000
L Superior occipital cortex	−10, −84, 22	6	3.52	4.25	0.0002
R Superior frontal gyrus	22, −4, 58	4	3.43	4.10	0.0003
R Middle frontal gyrus	34, 12, 42	13	3.35	3.98	0.0004
R Middle frontal gyrus	42, 20, 50	*	3.23	3.79	0.0006
R Precentral gyrus	38,−4,46	*	3.22	3.77	0.0006
L Middle frontal gyrus	−38, 48, 6	5	3.25	3.82	0.0005

Note. *E* = exposure, *S* = self-verbalization, *B* = breathing. * = a cluster with previous area emerged.

## Data Availability

The data presented in this study are available in the Supplementary Materials.

## Supplementary Materials

The following supporting information can be downloaded at https://drive.google.com/drive/folders/1wVOdPCafjJWesA_iqVgl84rffCCfrWad?usp=sharing (accessed on 25 July 2022).
